# Pulmonary Sequestration: An Unusual Cause for Massive Hemoptysis in an Adult Woman

**DOI:** 10.7759/cureus.6145

**Published:** 2019-11-13

**Authors:** Sanjeev Jadhav, Sachin Sanagar, Haridas Munde, Mona Jadhav, Jayalakshmi Kutty

**Affiliations:** 1 Cardiothoracic and Vascular Surgery, Apollo Hospitals, Navi Mumbai, IND; 2 Anesthesia, Apollo Hospitals, Navi Mumbai, IND; 3 Anesthesia, Jehangir Hospital, Pune, IND; 4 Pulmonology, Apollo Hospitals, Navi Mumbai, IND

**Keywords:** pulmonary sequestration, hemoptysis, thoracotomy

## Abstract

Pulmonary sequestration is a congenital anomaly characterized by nonfunctional lung parenchymal tissue receiving blood supply from systemic arteries instead of pulmonary arteries. It is a rare entity, and diagnosis is often missed given the condition’s presentation mimics other pulmonary diseases. Pulmonary sequestration leads to recurrent episodes of pneumonia, frequent hospital admissions, and, very rarely, fatal hemoptysis. Sometimes, pulmonary sequestration is diagnosed in adulthood when the patient presents with severe symptoms. We report the case of a 34-year-old woman with intralobar sequestration whose symptoms manifested in adulthood as occasional hemoptysis for two months followed by one episode of massive hemoptysis. Our case highlights the need for timely surgical intervention and thorough preoperative evaluation with imaging for optimal patient outcomes.

## Introduction

Pulmonary sequestration is a congenital anomaly in which nonfunctional lung parenchymal tissue is supplied with blood from systemic arteries rather than pulmonary arteries and usually by a branch from the thoracic or abdominal aorta in 75% to 80% of cases [1-2]. The diagnosis of pulmonary sequestration may be easily missed in adults, as many of the symptoms and the computed tomography (CT) manifestation overlap with other pulmonary pathologies such as lung cancer [1]. Pulmonary sequestrations comprise 0.15% to 6.4% of congenital lung malformations [3]. We report a case of intralobar pulmonary sequestrations (ILS) presenting in an adult woman with an episode of massive hemoptysis.

## Case presentation

**Figure 1:**
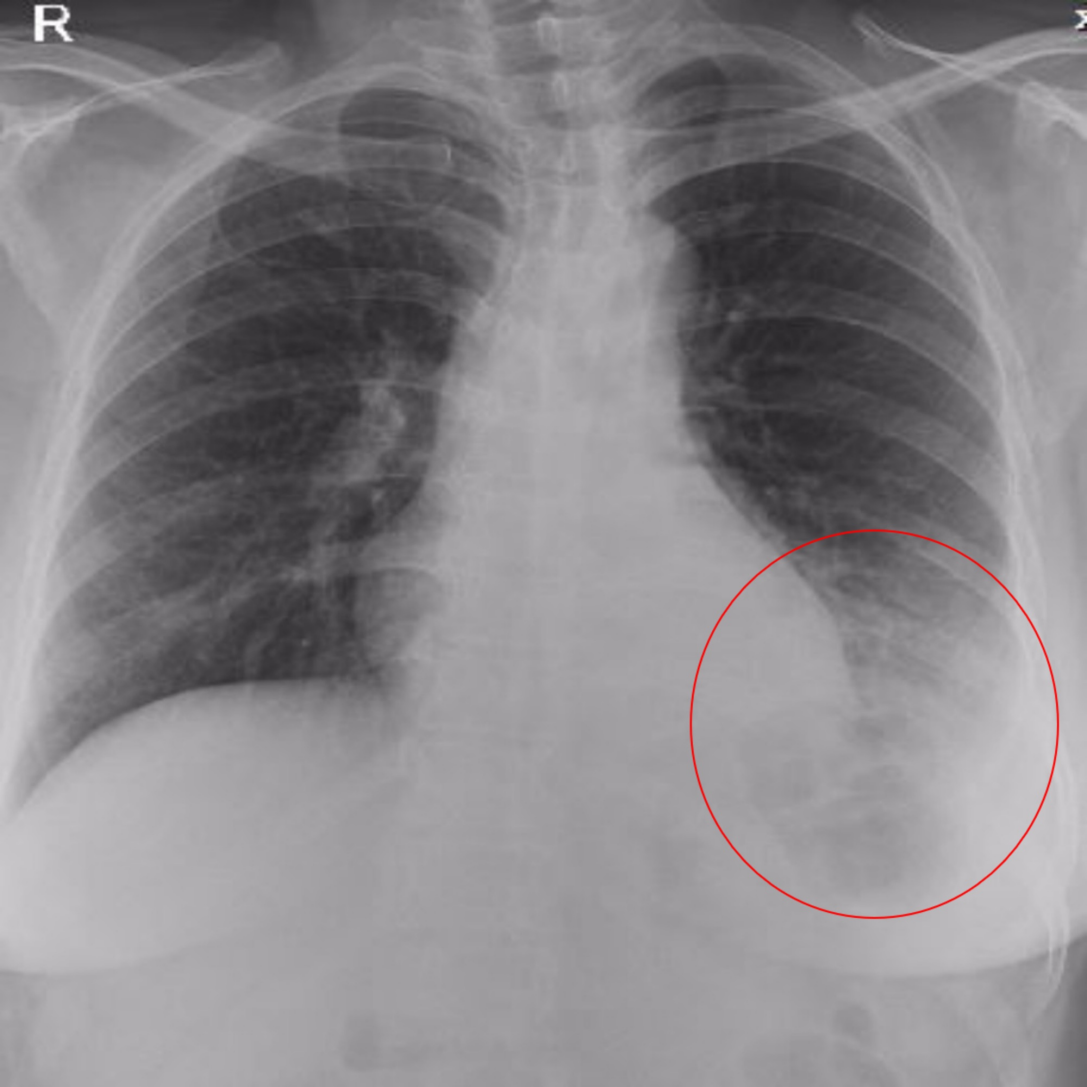
Chest roentgenogram showing diffuse opacity in the area of the left lower lobe

A 34-year-old woman presented with anorexia, frequent episodes of cough, foul breath, recurrent pneumonia, left lower back pain, and occasional hemoptysis lasting two months. These symptoms were unresponsive to antibiotics and all forms of medical management. Her hemoptysis frequency increased in the last week with one episode of massive hemoptysis. She had received complete antitubercular treatment for sputum acid-fast bacilli (AFB)-positive pulmonary tuberculosis five years prior to presentation. On clinical examination, her vital signs were unremarkable. Chest auscultation revealed reduced air entry in the left lower chest. The chest roentgenogram revealed an opacity in the area of the left lower lobe (Figure 1).

**Figure 2:**
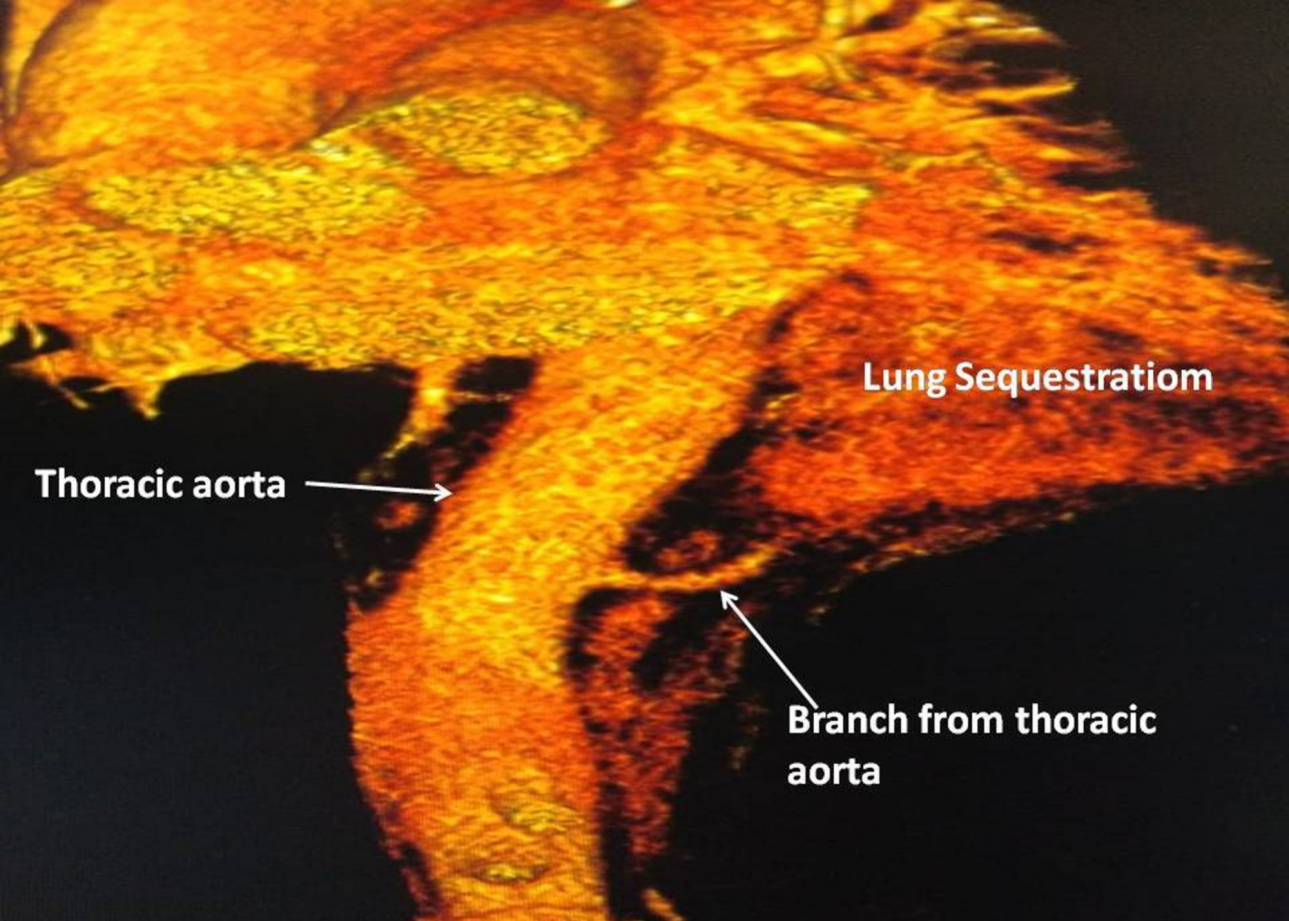
Feeding artery from the posterolateral thoracic aorta supplying to the sequestered lung

A computed tomography (CT) chest showed left lower lobe collapse-consolidation changes with an ill-defined heterogeneous area with nonenhancing hypodensities and cystic and necrotic changes in the absence of air bronchogram within. We noted a large artery arising directly from the posterolateral aspect of the thoracic aorta and supplying the left lower lobe mass (Figure 2).

Results of her hemogram, liver and renal function tests, and antitubercular antibody tests were unremarkable. Sputum culture was negative for tuberculosis. Bronchoscopy showed a normal trachea with no intraluminal mass. Bronchoalveolar lavage fluid was negative for malignant cells and AFB.

**Figure 3:**
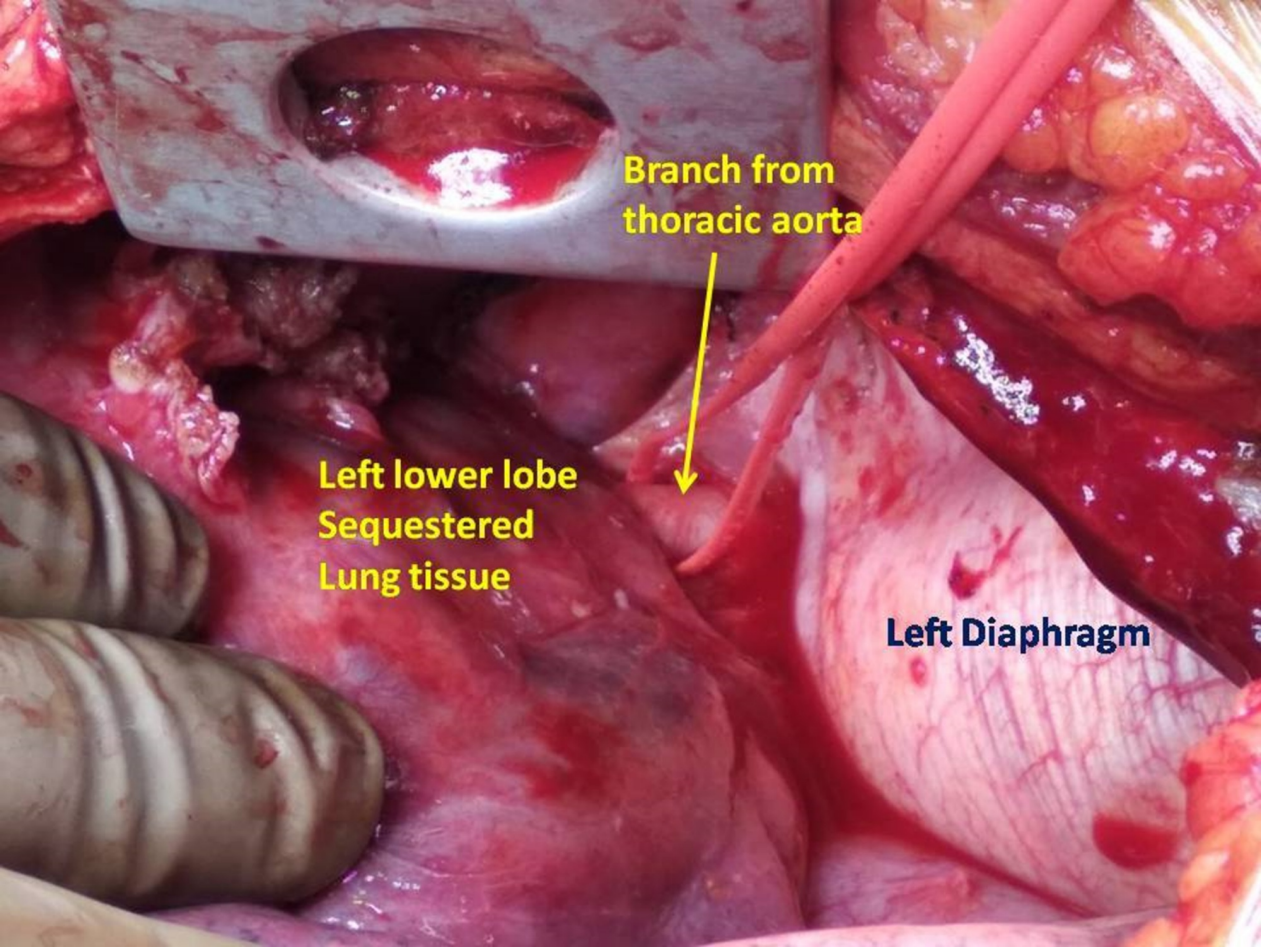
Intraoperatively looped feeding artery

After providing written informed consent, the patient underwent left lower lobectomy under general anesthesia by posterolateral thoracotomy approach. Intraoperatively, a large artery (8 mm) originating from the posterolateral thoracic aorta was ligated and divided, followed by left lower lobectomy (Figure 3). Intralobar sequestered lung tissue revealed blood and clots in the central cavity. Excised tissue was negative for gram stain, AFB culture, fungal culture, and GeneXpert test for tuberculosis (Cepheid, Inc., Sunnyvale, CA). Thoracic epidural analgesia was used as pain management for the initial two days. Her postoperative course was uneventful, and she was discharged on the fifth postoperative day.

**Figure 4:**
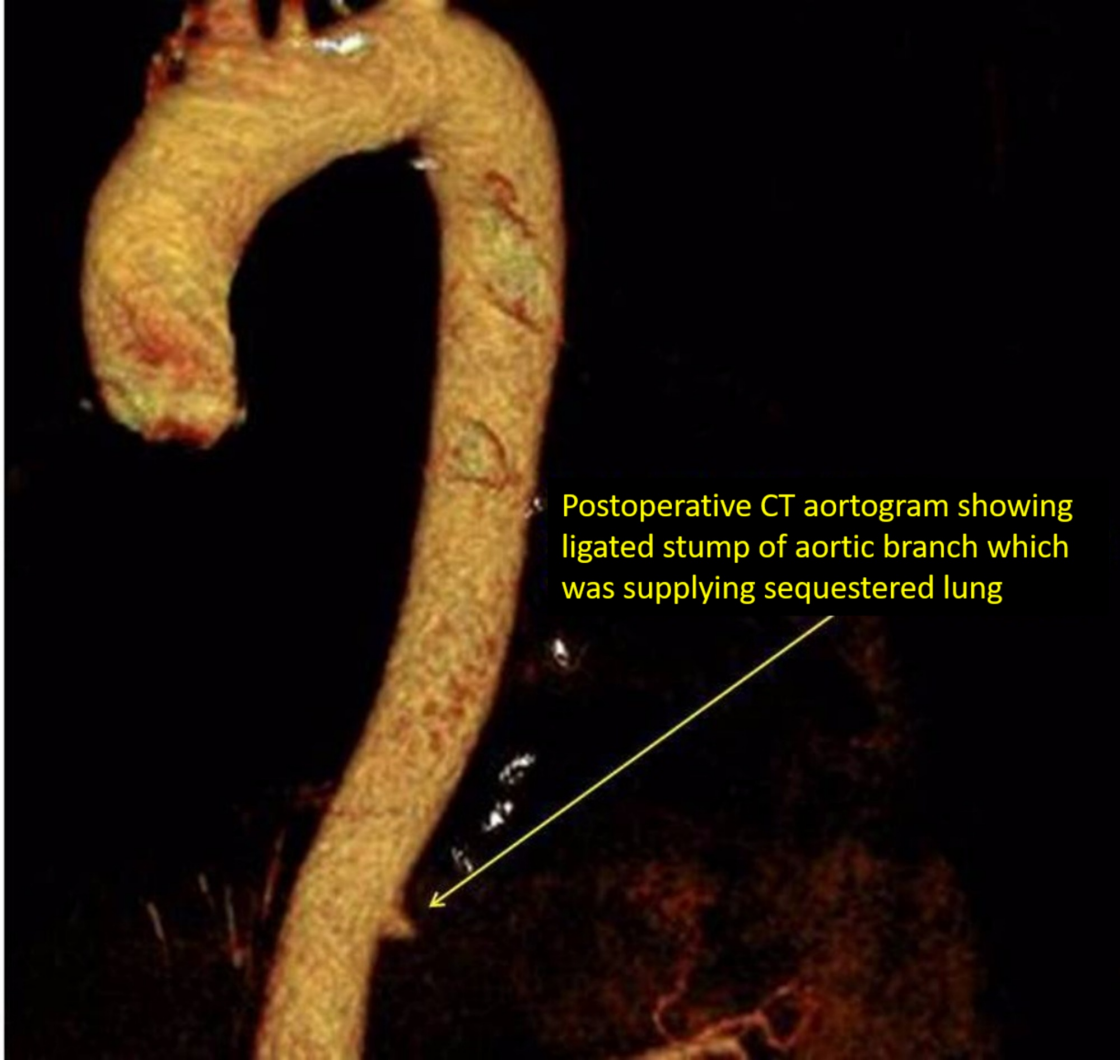
Postoperative CT aortogram showing the division of the feeding artery CT, computed tomography

Histopathology findings revealed congested benign lung parenchyma with cystic changes and chronic inflammatory exudates, fibrosis, and vascular sclerosis representing changes consistent with sequestration. Postoperative CT showed complete excision of the mass along with its arterial supply from the aorta (Figure 4). At her four-month follow-up evaluation, chest roentgenogram was unremarkable for the postoperative status and she was free from all pulmonary symptoms.

## Discussion

According to Kayhan, the pulmonary segment supplied by the systemic artery was first reported by Huber in 1877, which was later named "sequestration" by Pryce in 1946 [4]. Anatomically, sequestrations are classified as ILS (which is within a normal lobe without its own visceral pleura) and extralobar pulmonary sequestration (ELS, which is outside the normal lung and has its own visceral pleura).

ELS usually presents in the neonatal period as it is associated with other congenital anomalies such as hypoplasia of the lung. However, 50% of ILS cases present after age 20. Patients usually present with symptoms such as chronic cough, recurrent pneumonia, backache, chest tightness, and, very rarely, fatal hemoptysis [5]. ELS is 1.5 times more common in male patients. In nearly 66% of cases, ELS is seen in the left lower lobe [2]. Diagnosis is often missed as the symptoms and radiological findings overlap with other thoracic pathologies such as collapse consolidation, pleural effusion, and lung cancer. In our case, her one episode of massive hemoptysis prompted for high-resolution CT in which the feeding vessel was visualized, confirming the diagnosis of sequestration (and hence, CT aortography was not performed). Intraoperatively extensive adhesions of the sequestered lung with the chest wall were present. The feeding artery was ligated and divided, followed by left lower lobectomy. Postoperative CT aortogram showed a divided feeding branch at the thoracic aorta (Figure 4).

Surgical division of the feeding artery and lobar resection is the definitive treatment for ELS. Asymptomatic ILS should also be considered for surgical treatment due to the risk of massive hemoptysis and death [2]. Recently, cases treated by video-assisted thoracic surgery are reported. Recently, few reports of coil embolization for sequestration in an adult have been reported in the literature but lack long-term follow-up results. The overall prognosis after surgical treatment is good.

## Conclusions

Pulmonary sequestration should be considered a differential diagnosis of chronic undiagnosed pulmonary pathologies. Timely surgical intervention is a must to save the patient. Thorough preoperative evaluation with imaging and intraoperative surgical expertise leads to better outcomes in cases of pulmonary sequestration.
